# Determination of Clinical Process and Response Rate to Treatment in Patients with Gestational Trophoblastic Neoplasia (GTN) with Low and High Risk and Evaluation of Their First Pregnancy Outcome

**Published:** 2018-10-01

**Authors:** Mozaffar Aznab, Anisodowleh Nankali, Sara Daeichin

**Affiliations:** 1 Department of Internal Medicine, Taleghani Hospital, Kermanshah University of Medical Sciences, Kermanshah, Iran; 2 Department of Obstetrics and Gynecology, Imam Reza Hospital, Kermanshah University of Medical Sciences, Kermanshah, Iran

**Keywords:** Gestational trophoblastic neoplasia, Choriocarcinoma, High dose chemotherapy, Pregnancy

## Abstract

**Background:** The present study was conducted to determine the response to treatment in patients with GTN, the survival rate and to investigate the outcomes of first pregnancy after chemotherapy.

**Materials and Methods:** The treatment protocol was based on the FIGO Staging of GTN and the Modified WHO Prognostic Scoring.

**Results:** Complete remission was achieved with MTX in 100% of the low-risk patients and with combination therapy in 91% of the high-risk cases. Out of 27 low-risk patients, 21 had no metastasis 6 had lung metastasis, 18 preserved their fertility and conceived in the first year following the chemotherapy. Out of 3 patients who had developed invasive moles, 1 got pregnant after chemotherapy. Four of the patients with choriocarcinoma conceived in the first year following the chemotherapy. In the patient with placental site trophoblastic tumors, there was no pregnancy due to hysterectomy.

**Conclusion:** GTN was found to be a chemosensitive condition, but more effective therapeutic protocols are therefore required.

## Introduction

 Gestational Trophoblastic Neoplasia (GTN) is the only neoplasia that preserves the patient fertility after undergoing different medical and surgical treatment modalities. GTN is more common in Asia than in North America or Europe ^1^^,^^2^. GTN arises from the trophoblastic tissue and consists of six clinicopathological entities, including complete hydatidiform mole (CHM) and partial hydatidiform mole (PHM), invasive mole, choriocarcinoma(CCA ), placental site trophoblastic tumors (PSTT) and epithelioid trophoblastic tumor^3^. The incidence of CHM and PHM is 1 and 3 per 1000 pregnancies and 3 per 1000 pregnancies, respectively^4^. The incidence of (hydatidiform mole) HM after spontaneous miscarriage is estimated as 1:15000. Choriocarcinoma(CCA) can occur after any type of pregnancy; however, it most commonly develops after CHM^5^. The incidence of CCA in Southeast Asia and Japan is higher. An invasive mole (IM) develops in approximately 15% of patients with CHM and in about 1-15% of patients with PHM. The frequency of PSTT is less clear; however, tends to occur mostly after normal pregnancies or spontaneous miscarriages, although it makes up about 0.2% of the cases of GTN in the UK^6^ and originates from an interstitial trophoblast^7^^,^^8^^,^^9^. According to the FIGO Staging of GTN and the Modified WHO Prognostic Scoring, the GTN patients are classified into a low-risk group and a high-risk group and the candidates for single and combination chemotherapy were thus selected. For the low-risk patients receiving mono-chemotherapy, MTX is the preferred treatment. The high-risk patients typically receive combination chemotherapy. In our center, we prescribe combination chemotherapy to low-risk group with lung metastasis^10^, and also in the presence of a high number of metastases in lung or high β-HCG^11^. This is to prevent resistance to mono therapy. The majority of the high-risk patients with GTN may present with many metastases months or years after the causative pregnancy. Different multi-drug therapies have been developed for these patients such as MAC, CHAMOCA and EMA-CO^12^. The multi-agent CT EMA/CO has become widely accepted as the treatment of choice for high-risk patients. EMA/CO may improve the primary response rate and lead to a lower acute toxicity rate compared to the MAC regimen, especially among high-risk patients. The present study was conducted to explore the epidemiology, management and treatment outcomes of patients with persistent GTN in Iran, and to evaluate the patients’ experiences of pregnancy after chemotherapy. After completion of their chemotherapy, the women were asked about their pregnancy wishes, fertility, pregnancies and pregnancy outcomes, menstrual cycle, use of contraception and menstrual cycle.

## MATERIALS AND METHODS

 The current cross-sectional study is based on medical records of patients with gestational trophoblastic neoplasia that registered in hospitals of Kermanshah University of Medical Sciences, Iran from January 2006 to 2015. A total of 44 patients were included in the study. According to the inclusion criteria, patients were divided into two groups in this study. The first group consisted of patients diagnosed with GTN, following a molar evacuation and according to the FIGO guidelines and the second group included patients with increased titers (β-HCG) without a history of molar pregnancy, those with a history of abnormal bleeding in the reproductive age or evidence of metastasis and those who developed rising β-HCG titers in the examination to rule out gestational trophoblastic neoplasia (β-HCG). A total of 44 patients were examined in terms of age, parity, clinical picture, pretreatment β-hCG, histopathology, radiological findings, type of trophoblastic disease, type of surgical treatment, type of chemotherapy, response to treatment and mortality associated with this disease. All the patients were staged according to the current International Federation of Gynecology and Obstetrics (FIGO) Staging of GTN and the Modified WHO Prognostic Scoring System. Upon admission, all the patients underwent a pretreatment evaluation to determine the extent of their disease; their blood pressure was then measured before they underwent a laboratory evaluation (CBC), coagulation studies, thyroid, renal and hepatic function. Furthermore, a speculum examination was performed to identify cases of vaginal metastasis.

 Brain, lung, pelvic and abdominal scans were also performed on the patients. According to the FIGO scores and clinicopathological profiles, the patients with a low risk score less than 7 who showed no metastasis were given single chemotherapy^13^^,^^14^ with a high dose infusion of methotrexate with folic acid rescue (100 mg/m^2^ IV push followed by 200 mg/m^2^ IV 12-hour folic acid rescue for three doses beginning 12 hours after starting the MTX administration) and repeated their therapy every two weeks as required; however, if β-HCG levels remained constant or if MTX toxicity occurred, the chemotherapy was substituted with an alternative drug^13^^,^^14^. Patients with a low-risk sore less than 7, positive lung metastasis and a FIGO score above 7, or with a high-risk score and resistance to sequential single-agent chemotherapy initially treated with multi-agent chemotherapy protocol (EMA-CO). The course of chemotherapy continued until β-HCG levels reached normal. All the patients received at least one additional course of chemotherapy (consolidation) if no drug toxicity occurred. For those with invasive mole, chemotherapy was the preferred method of treatment, although hysterectomy was also considered. Hysterectomy was the preferred method of treatment for PSTT^15^. The patients were advised not to become pregnant for at least one year after their treatment; i.e. to keep their method of contraception until 6 months following the completion of their chemotherapy and/or their first normal titer. The patients were recommended to take oral contraceptives as they cause the suppression of endogenous LH, which may interfere with the measurement of HCG at low levels^16^^,^^17^^,^^18^.

To avoid toxicity, leucovorin was administered to treat the potential toxic effects of MTX overdose. The toxicity of the EMA-CO chemotherapy was evaluated. Stem cell support with Granulocyte Colony Stimulating Factor (G-CSF) was carried out to avoid dose reduction and treatment delays during the administration of EMA-CO. After completion of chemotherapy, serum hCG levels were measured at one-month intervals for a whole year. Physical examinations were also performed at intervals of 3 months. Other tests such as x-rays were also performed at intervals of 3 months for a whole year. The patients kept their method of contraception during the treatment and one year after the completion of the chemotherapy^18^. 

## Results

 The main outcomes were measured in terms of age, gravidity, level of β-hCG, treatment, follow-up, mortality and pregnancy outcome. Table1 presents the FIGO scores and the clinicopathological profiles of our patients. 

**Table 1:** FIGO scoring and clinicopathologic profile of our patients with GTN

**GTN**	**Risk Groups**												**N**					**Percent**			
Clinicopathological entities	LOW RISK												30 cases					68.2%			
	HIGH RISK												14 cases					31.8%			
Age	< 20 yr				8 cases				21-30 yr	19 cases			31-40 yr	11 cases				> 40 yr	6 cases		
Antecedent pregnancy	Molar Pregnancy							22	Term Pregnancy				Abortion					unknown			
	cases								2 case				6 case								
hCG level (IU/liter)	<1000	23					(52%)		1,000- 10,000		9		10,000-100,000			10 (22.5%)		>10 0,000			2(4.5% )
	case								(20.5% ) case				cases					cases			
Gravity	Gravidity : 1					(11			Gravidity : 2 (17cases)				Gravidity : 3				(5 cases)	Gravidity : 4			(4 cases )
	cases )																				
stage I	21 patients																				
stage II	6																				
stage III	16(Including 10 pts CCA and 6 pts low risk with lung metastasis																				
stage IV	1 pts CCA																				

All the patients were classified into two risk groups based on the FIGO Staging of GTN and the Modified WHO Prognostic Scoring System; the low-risk patient group included 21 cases with low-risk GTN (risk score < 7) and no metastasis as well as 6 cases with low-risk GTN (risk score < 7) and metastasis; the high-risk patients included all the patients with prognostic scores > 7, consisting of 11 patients with CCA, invasive mole (n=3) and PSTT (n=3). According to the FIGO staging, of 44 patients examined, 21 were in stage I, 6 in stage II, 16 in stage III and 1 patient was in stage IV. The participants ranged from 16 to 46 in age and had a mean age of 29 years. The highest frequency of GTN was observed in the 21-30 age group (N=18). In 22 of the cases, the condition was preceded by a molar pregnancy; only in a minority, it was preceded by a miscarriage (n=6) or term gestation (n=2). In other cases, there was no specific history of pregnancy, abortion and other complications related to pregnancy. 

Vaginal bleeding was the most common clinical presentation (92.5%) and only 1 patient presented with malignant hypertension. A large theca lutein cyst with pleural effusion was observed in one of the patients. Most of the 22 cases with a history of molar evacuation were primigravida (n=17/22) and only 1 patient had a previous history of molar pregnancy. Five out of the 44 patients had undergone hysterectomy. The indications of hysterectomy included 3 cases of PSTT, 1 case of invasive mole with uterine perforation and 1 case of choriocarcinoma. One patient with invasive mole presented with acute abdominal pain and signs of hemoperitoneum, and thus underwent hysterectomy. She received multi-agent chemotherapy (EMA-CO) and ultimately showed a good response. Of the 43 women who had remission after the treatment, 7 were followed-up less than one year, 19 for one to three years and 17 for more than three years. One woman with choriocarcinoma was followed-up less than one year before she died. Of the 43 women who had remission, the menstrual status of 27 who were in the low-risk group was evaluated and their overall menstrual status occurred. All the low-risk patients with a risk score less than 7 showing no metastasis (n=21) and receiving a single-agent treatment, in addition to all 6 low-risk patients with a risk score less than 7 showing metastasis or with stage III of the disease according to their clinicopathological profile and receiving EMA-CO treatment were still in complete remission after the treatment (100% rate of remission). In the 11 patients with choriocarcinoma, the response to treatment with EMA-CO was 90%, and 1 patient in this group received a second-line of chemotherapy, but died due to disease recurrence in the brain. Two out of the 3 cases of invasive mole received single-agent chemotherapy and showed a good response. Three patients with PSTT underwent surgical treatment, received single-agent chemotherapy and showed a good response (Table 2).

**Table 2:** Treatment, outcome and deaths per histological subtype for our patients with GTN

**Risk Group**	**Number patients**	**Treatment**	**Response Rate**	**Live (%)**	**Death (%)**
low-risk GTN (risk score < 7)	21 case	Single-agent chemotherapy	complete remission	100%	-
low-risk GTN (risk score < 7) with metastasis or stage III	6 cases	EMA-CO chemotherapy	complete remission	100%	-
High-risk GTN (risk scores > 7)		EMA-CO chemotherapy			
Choriocarcinoma(CCA)	11 cases	EMA-CO chemotherapy	91%	91%	9%
Invasive mole	3 cases	Single-agent treatment(2 cases), EMA-CO treatment(one case with perforation	complete remission	100%	
PSTT	3 cases	single-agent chemotherapy & surgery	complete remission	100%	

The toxicity of the MTX chemotherapy was evaluated. The dose-limiting toxicities of MTX included bone marrow suppression and gastrointestinal toxicity. Moreover, the most common toxicity was hematologic and included leukopenia, anemia and thrombocytopenia. GI toxicity manifested in the forms of mucositis, nausea and vomiting. Alopecia was the most common toxicity in the present study. The toxicity of the EMA-CO chemotherapy was evaluated and alopecia, nausea and myelosuppression (neutropenia and thrombocytopenia) were observed. The EMA-CO regimen was generally well tolerated.

**Overall Survival**

The mean follow-up was 4.84 yr. The overall survival in lower risk patients (score <7) with and without of metastasis was 100%. The overall survival in patients with invasive mole and PSST was 100%. The overall survival in the high-risk group included patients with metastasis was 90%. 

**Pregnancy outcomes**

The researchers then evaluated subsequent pregnancies one year after remission (Table 3). In the group of patients with CCA, 2 were older and their age was no longer suitable for pregnancy, 4 were over 48 and did not want to get pregnant, and 1 had undergone hysterectomy. Of the remaining patients in the choriocarcinoma group, 5 were in the age group of 18-25, and, after two years of treatment, they wanted to get pregnant and showed good outcomes. Four out of the 6 patients with a low risk score > 7, positive metastasis or with clinicopathological stage III disease during the follow-up showed good pregnancy outcomes. One of these patients has since been pregnant twice. After two years, of the 3 patients with invasive mole, one wanted to become pregnant and successfully conceived, one did not want to get pregnant, and 1 had undergone hysterectomy. Of the 21 patients with a low risk score < 7 and no metastasis, 14 wanted to become pregnant and successfully conceived and had a good pregnancy outcome. The pregnancy outcomes included 18 full-term live births (n=18), premature deliveries (n=4), still birth (n=1) and abortion (n=1); no ectopic pregnancies were observed (Table 3). A total of 39% of these patients conceived successfully within the first year after the doctors had permitted their pregnancy and 59% became pregnant in the second year. The method of delivery in 67% of the patients was normal vaginal delivery, while the remaining patients underwent C-section. Fetal abnormalities were not observed in any of the cases and all the babies were born alive.

**Table 3:** outcome of pregnancy in our patient with GTN

**Risk group** **(number patients)**	**N.P**	**No wish pregnancy**	**No suitable for** **pregnancy (older** **age)**	**Pregnancy (n)**	**Perinatal** **outcomes: n**	**No suitable for** **pregnancy** **(hysterectomy)**
Choriocarcinoma (11)	11	4	2	5 (age 18-25yr)	Premature: 1 patients Term live births: 4	1
Low risk < 7 with metastasis (6)	6	1	1	4	Abortion: 1 patients Term live births: 3	
Invasive mole (3)	3	1	1(hysterectomy)	1	Term live births: 1	
Low risk < 7, no metastasis (21)	21	5	2	14	Still birth: 1 patients Premature: 3patients Term live births: 10	
PSTT	3	-	3(hysterectomy)	0		3

**Follow-up after the treatment**

The risk of disease recurrence one year after the patient achieved remission was less than 1%. It is necessary to advise the patients to use a reliable form of hormonal contraception during the first year of remission. Due to the 1% risk for the development of a second mole in subsequent pregnancies, an early ultrasound is recommended for all future pregnancies. The patients are advised not to become pregnant for at least one year after completing the chemotherapy as pregnancies may make it difficult to recognize relapses. 

## Discussion

 The incidence of GTN differs by geographical location. GTN is a highly curable group of pregnancy-related tumors that are highly chemosensitive. The optimal management of gestational trophoblastic disease depends on the early diagnosis of the disease, the correct stratification of the risk category and the performance of appropriate treatments with different modalities such as chemotherapy and surgery. The early diagnosis of the disease and its proper management affect the outcome of GTN significantly, which is a potentially curable condition, even in a disseminated state. Single-agent chemotherapy and intermediate-dose MTX infusional chemotherapy tend to show very good responses in patients with the non-metastatic and low-risk form of the disease if carried out appropriately and if no significant toxicities occur; a 100% rate of recovery was observed in this study with these treatments. The EMA-CO regimen is the most widely used first-line combination chemotherapy for high-risk GTN patients and for methotrexate-resistant or recurrent GTN cases and also in our study for those with a low-risk score < 7 and metastasis^10^^,^^11^. After their hCG levels returned to normal, the patients underwent additional chemotherapy courses to avoid the recurrence of the disease. Nevertheless, approximately 25–30% of the cases of GTN are resistant to treatment or will relapse after their initial chemotherapy^19^. In this study, the highest frequency of GTN was observed in the 21-30 age group with a mean age of 29 years. A total of 60% of the patients with choriocarcinoma were in the age group of 18-25, although other studies reported a lower average age for these patients. Similar to other studies conducted on the subject, the lung was the most common site of metastasis. In other studies, complete remission was observed in 96% of the low-risk patients and a 70-90% remission rate was observed in the high-risk group receiving EMA-CO^20^; in the present study, a 94.5% rate was observed in those receiving the EMA-CO treatment and 100% rate in the low-risk group. Of the total of 44 patients examined in this study, 43 are still alive. Complications caused by the administered drugs were mainly hematologic and mucosite, and febrile neutropenia was also observed in the patients who received the EMA-CO regimen. Preserving fertility is a critical issue in high-risk cases of GTN, and since this tumor frequently occurs in women less than 30 years of age, the affected person often wants to become pregnant after the completion of their chemotherapy. Some researchers have found no relationships between the type of chemotherapy regimen and the pregnancy outcome^21^^,^^22^, and the chemotherapy agent used has no effects on pregnancy. Rustin GJ et al.^23^ reported that women receiving combination chemotherapy are less likely to conceive or have a live birth compared to those receiving only methotrexate. Chemotherapy can affect the ovarian function. Newlands ES^24^ showed that the majority of women established regular menstruation within six months after completing their therapy. As a result, treating gestational trophoblastic disease with chemotherapy is compatible with the preservation of fertility. If the GTN patient receives a chemotherapy agent that is selectively toxic to rapidly dividing cells such as developing follicles in the ovaries, her conceivability or the generation of the fetus may be affected and pregnancy outcomes are therefore controversial in this group. However, several studies have reported that chemotherapy does not influence later pregnancies. Woolas et al, (1998) observed no differences in conception rates or pregnancy outcomes between patients who were treated with single-agent MTX and those who received multiple-agent chemotherapy. The present study showed that the rates/likelihood of term and preterm delivery, still birth, abortion, ectopic pregnancy and congenital anomalies in former GTN patients do not differ from the overall average rates. Other findings also suggest that pregnancy following treatment for GTN not lead to outcomes that differ from the pregnancy outcomes observed in the healthy population. 

## CONCLUSION

 The treatment of patients with GTN was successful but associated with a high rate of remission; however, these patients can be assured that their post-treatment pregnancies can still have favorable outcomes.
